# EVEscape: Revealing potential escape sites based on the viral variation landscape

**DOI:** 10.52601/bpr.2024.240902

**Published:** 2024-04-30

**Authors:** Yaling Li, Aiping Wu, Hang-Yu Zhou

**Affiliations:** 1 Zhejiang Lab, Hangzhou 31121, China; 2 State Key Laboratory of Common Mechanism Research for Major Diseases, Suzhou Institute of Systems Medicine, Chinese Academy of Medical Sciences & Peking Union Medical College, Suzhou 215123, Jiangsu, China

The continuous mutation and evolution of viruses to evade the host immune response present a formidable challenge to combating infectious diseases. Accurate prediction of viral immune escape sites is crucial for disease diagnosis, therapy development, vaccine design, and other strategies aimed at combating emerging infectious diseases. During the COVID-19 pandemic, remarkable progress has been made in sequencing technology and infrastructure, leading to an unparalleled accumulation of genomic data specifically pertaining to a singular pathogen. This advancement has also stimulated innovative approaches in data-driven research methodologies (Obermeyer *et al*. 2022; Maher *et al*. 2022; Hie *et al*. 2021).

Experimentally, the conventional approaches for investigating virus mutation encompass reverse genetics methods such as pseudovirus mutagenesis experiments and the emerging deep mutational scanning (DMS). Nevertheless, these methods are often resource-intensive and time-consuming. Furthermore, their capacity to explore the extensive range of potential variants is relatively limited.

In a recent study focused on the spike protein of the SARS-CoV-2 virus, although DMS has the capacity to evaluate the biological effects arising from up to 300,000 potential mutation combinations (Dadonaite *et al*. 2023), this number still falls short when contrasted with the exponential mutation possibilities that the entire spike protein presents. Additionally, DMS methods are reliant on the underlying genetic background of mutant strains. In recent years, various artificial intelligence algorithms have been proposed for predicting virus mutations, including Bayesian hierarchical regression (Obermeyer *et al*. 2022), logistic regression (Maher *et al*. 2022), and Bi-directional Long Short-Term Memory (BiLSTM) neural networks (Hie *et al*. 2021), *etc*. The common limitation of these approaches lies in their dependence on extensive sequencing data and epidemiological information from established pathogens, thereby failing to offer timely and efficacious prior knowledge for vaccine design in the initial stages of emerging infectious diseases.

In a recent study, Marks' team proposed EVEscape (Thadani *et al***.** 2023), an integrated model that combines deep learning with biophysical constraints to enhance its predictive capabilities. The EVEscape model is derived from the previously proposed mutation effect evolution model, known as EVE (Frazer *et al*. 2021), by Marks' team. The core of EVE is a Bayesian variational autoencoder that classifies and scores the pathogenicity caused by amino acid substitutions in human proteins based on protein sequence homology. After the COVID-19 outbreak, Marks' team expanded the EVE model to create the EVEscape model, which incorporates virus fitness metrics, antibody accessibility, and antibody binding in order to predict potential viral escape. By leveraging extensive historical viral sequence data and simulating viral protein evolution processes, the EVEscape model constructs a comprehensive feature space of virus mutations and proficiently predicts potential mutation trajectories that viruses may adopt under immune pressure (Fig. 1). Compared to previous prediction methods, the EVEscape model offers several key advantages:

**Figure 1:**
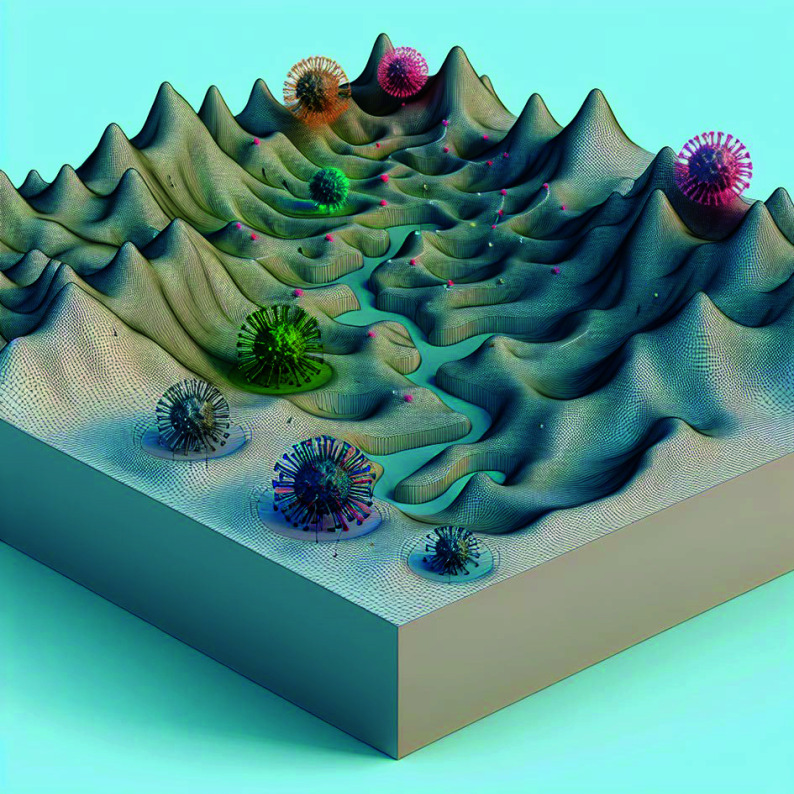
The evolutionary landscape of virus constrains the possible mutation space of virus. The figure was produced using the DALL-E 3 generative model in response to the prompt "draw viruses in an evolution mesh landscape". From a set of 50 generated images, the selected depiction was chosen for its illustrative capture of the evolutionary landscape's influence on — and limitations imposed upon — viral forms

(1) The EVEscape model focuses on the inherent mutational potential of the pathogen itself. It utilizes historical mutation data of the same kind of the pathogen, rather than relying on real-time virus information, thereby rendering it suitable for both early-stage virus outbreaks and continuous assessment of novel variants.

(2) By analyzing the intrinsic factors of pathogen escape, the EVEscape model additionally introduces biophysical and structural biology knowledge (such as protein exposure and hydrogen bond formation potential) as constraints to screen the generated sequences to ensure that the predicted mutations are structurally stable and functionally reasonable. This integrated approach makes the prediction more accurate and comprehensive.

(3) The EVEscape model exhibits a high level of prediction accuracy. In the retrospective analysis of SARS-CoV-2 mutation prediction, EVEscape demonstrates a prediction accuracy exceeding 85%, which is comparable to widely employed high-throughput experimental methods such as DMS.

(4) The EVEscape model provides a general method for virus escape prediction, which can be extended to diverse virus types. As an illustration, the EVEscape model effectively captures pivotal mutations in previously understudied Lassa and Nipah viruses.

In summary, the EVEscape model offers a scalable computational approach that can be employed to forecast escape mutations in both the early and subsequent stages of viral evolution during a pandemic. More importantly, the method reveals a viable approach to narrow down the scope of mutation analysis, thereby rendering virus mutation prediction within reach. In practical applications, the integration of the model with real-time sequence data or antibody data holds the potential for further enhancing predictive efficacy. The prediction of viral escape mutations can provide valuable guidance for the development of public health measures and the allocation of medical resources, aiming to effectively mitigate the human and economic impact caused by infectious disease epidemics.

## Conflict of interest

Yaling Li, Aiping Wu and Hang-Yu Zhou declare that they have no conflict of interest.
